# Supplementary material for: Testing the psychometric properties of the Swedish version of the EPOCH measure of adolescent well-being
Source: PLoS One. 2021 Oct 29;16(10):e0259191. doi: 10.1371/journal.pone.0259191 (PMC8555806; doi:10.1371/journal.pone.0259191)
Supplement: S1 Appendix — (DOCX) [file pone.0259191.s001.docx]

**EPOCH Measure of Adolescent Well-being (Kern, Waters, Adler & White 2015)**

**In Swedish –på svenska**

Frågorna gäller hur du har haft det de senaste 6 månaderna.

| **Item** |
| --- |
| 1. När någonting trevligt händer mig, finns det människor jag gärna berättar det för. |
| 1. När jag påbörjat något slutför jag det alltid. |
| 1. Jag är optimistisk om min framtid. |
| 1. Jag känner mig glad. |
| 1. När jag håller på med något, blir jag så engagerad i det att jag tappar bort tiden. |
| 1. Jag har ofta roligt. |
| 1. Jag blir helt uppslukad av det jag gör. |
| 1. Jag älskar livet. |
| 1. Jag håller på med mina läxor tills jag är helt klar med dem. |
| 1. När jag har problem av något slag, har jag människor som jag kan vända mig till. |
| 1. Jag går så upp i det jag gör att jag glömmer allt annat. |
| 1. När jag håller på att lära mig något nytt glömmer jag bort tiden. |
| 1. I osäkra tider förväntar jag mig det bästa. |
| 1. Det finns människor i mitt liv som verkligen bryr sig om mig. |
| 1. Jag tror att det kommer att gå bra för mig. |
| 1. Jag har vänner som jag verkligen bryr mig om. |
| 1. När jag bestämt mig för att göra något håller jag fast vid min plan. |
| 1. Jag tror att saker löser sig, oavsett hur svårt det kan verka. |
| 1. Jag är en som jobbar hårt. |
| 1. Jag är en glad typ. |
